# Supplementary material for: Bioinformatics-based prediction of conformational epitopes for human parechovirus
Source: PLoS One. 2021 Apr 1;16(4):e0247423. doi: 10.1371/journal.pone.0247423 (PMC8016246; doi:10.1371/journal.pone.0247423)
Supplement: S1 Table — (DOCX) [file pone.0247423.s001.docx]

**S1 Table.** Comparison of our and Borley *et al*.’s prediction results for FMDV.

| VPs | Secondary structure  of known epitopes | Borley's results | | | | |  | Our results | | | | |
| --- | --- | --- | --- | --- | --- | --- | --- | --- | --- | --- | --- | --- |
|  |  | O1K | Reduced O1K | A | C | SAT-1 |  | O | A | C | SAT-1 | SAT-2 |
| VP1 | BC loop | ＋ | ＋ |  |  | ＋ |  | ＋ | **＋^a^** | **＋** | ＋ | ＋ |
|  | DE loop | ＋ |  | ＋ | ＋ |  |  | ＋ | ＋ | ＋ | **＋** | ＋ |
|  | HI loop | ＋ | ＋ | ＋ | ＋ |  |  | ＋ | ＋ | ＋ | **＋** | ＋ |
|  | C-terminus | ＋ | ＋ | ＋ | ＋ | ＋ |  | ＋ | ＋ | ＋ | ＋ | ＋ |
| VP2 | BC loop | ＋ | ＋ | ＋ | ＋ | ＋ |  | ＋ | ＋ | ＋ | ＋ |  |
|  | EF loop |  |  |  | **＋** | **＋** |  |  |  |  |  |  |
|  | HI loop | ＋ | ＋ | ＋ | ＋ | ＋ |  | ＋ | ＋ | ＋ | ＋ | ＋ |
| VP3 | N-terminus |  | ＋ |  |  | ＋ |  |  |  |  | ＋ |  |
|  | BC loop | ＋ | ＋ | ＋ | ＋ | ＋ |  | ＋ | ＋ | ＋ | ＋ | ＋ |
|  | EF loop |  |  |  |  | ＋ |  | **＋** | **＋** | **＋** | ＋ | ＋ |
|  | HI loop |  |  | ＋ | ＋ | ＋ |  | **＋** | ＋ | ＋ | ＋ |  |
|  | C-terminus |  |  |  | ＋ | ＋ |  |  | **＋** | ＋ | ＋ | ＋ |
