# Supplementary material for: Bioinformatics-based prediction of conformational epitopes for human parechovirus
Source: PLoS One. 2021 Apr 1;16(4):e0247423. doi: 10.1371/journal.pone.0247423 (PMC8016246; doi:10.1371/journal.pone.0247423)
Supplement: S2 Table — (DOCX) [file pone.0247423.s002.docx]

**S2 Table.** The sequence conservation of VP3-91, -92 and VP0-257 among HPeV1-19.

| Serotypes | VP3-91 | VP3-92 | VP0-257 |
| --- | --- | --- | --- |
| HPeV1 (n=57) | N (100%) | H (100%) | S (100%) |
| HPeV2 (n=3) | N (100%) | H (100%) | S (100%) |
| HPeV3 (n=130) | S (100%) | S (100%) | A (100%) |
| HPeV4 (n=12) | S (100%) | S (100%) | A (100%) |
| HPeV5 (n=9) | S (100%) | S (100%) | A (100%) |
| HPeV6 (n=7) | T (100%) | H (85.7%),Y (14.3%) | I (100%) |
| HPeV7 (n=1) | S (100%) | S (100%) | A (100%) |
| HPeV8 (n=3) | S (100%) | E (100%) | S (100%) |
| HPeV14 (n=5) | S (100%) | H (100%) | A (100%) |
| HPeV17 (n=6) | S (100%) | S (83.3%),H (16.7%) | A (83.3%),S (16.7%) |
| HPeV18 (n=1) | S (100%) | S (100%) | A (100%) |
| HPeV19 (n=1) | G (100%) | S (100%) | S (100%) |
