# Supplementary figures and images for: Bioinformatics-based prediction of conformational epitopes for human parechovirus
Source: PLoS One. 2021 Apr 1;16(4):e0247423. doi: 10.1371/journal.pone.0247423 (PMC8016246; doi:10.1371/journal.pone.0247423)

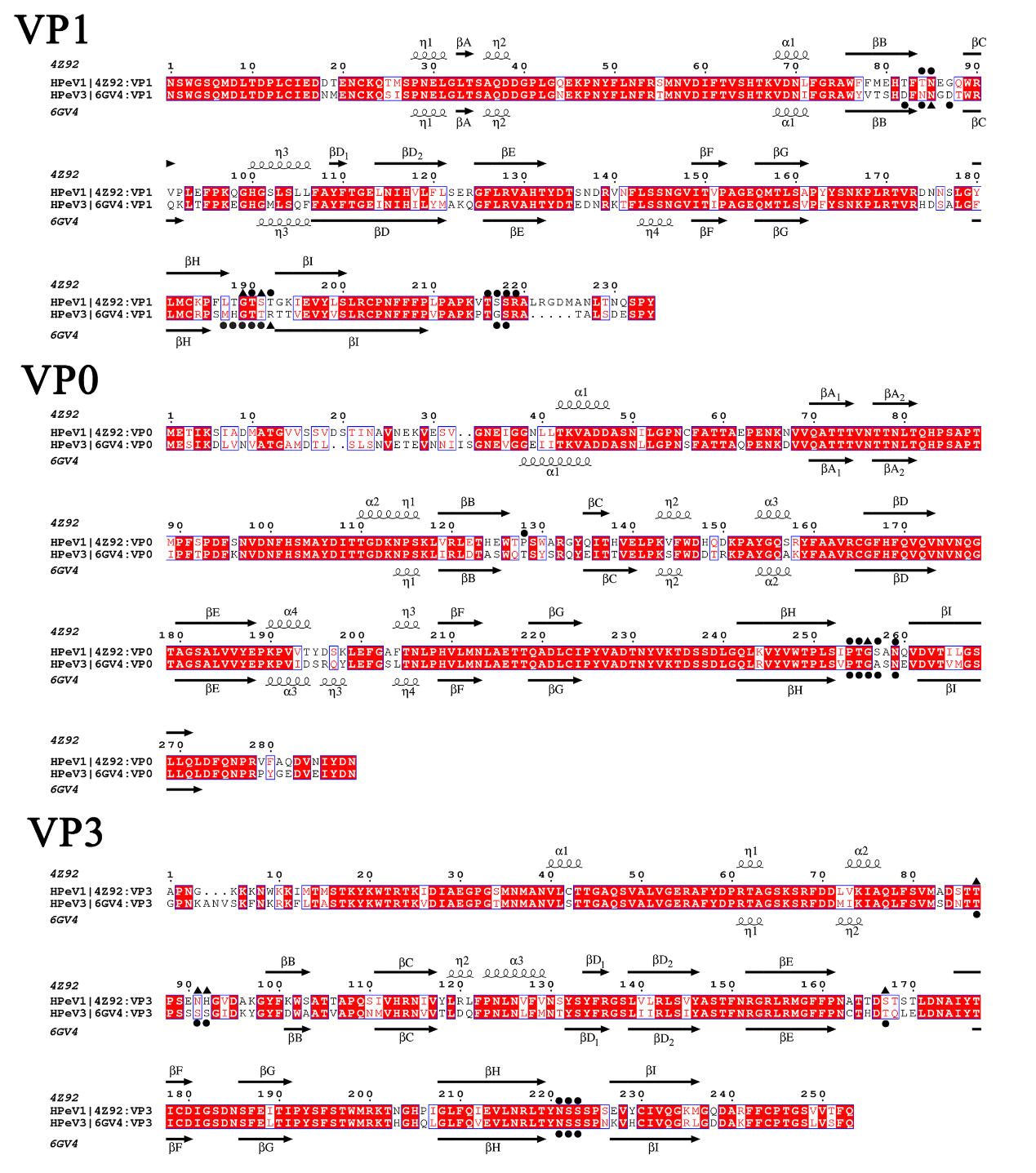


**S1 Fig.** The sequence alignment of VP1, VP0 and VP3 of HPeV1 and HPeV3.

Supplement: S1 Fig — The secondary structure elements and predicted epitopes from the HPeV1and HPeV3 are shown on the corresponding protein sequence alignment as β-sheets (arrows), α-helices (spirals), and the predicted core epitopes (black circles) and surrounding epitopes (black triangles). Sequence annotations on the left side represent to the virus genotypes and corresponding VP. Sequence identities between HPeV1 and HPeV3 are colored on a scale of white (no identity) to red (full identity). (DOCX) [file pone.0247423.s003.docx]

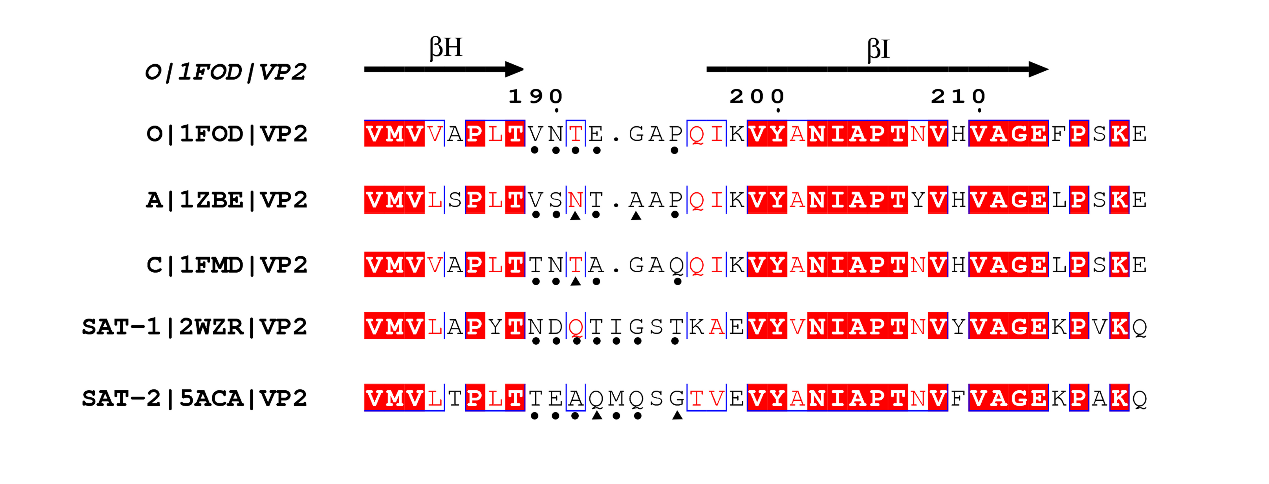


**S2 Fig.** The schematic depiction of core epitopes and surrounding epitopes.

Supplement: S2 Fig — The symbols used in this figure are the same as S1 Fig. (DOCX) [file pone.0247423.s004.docx]

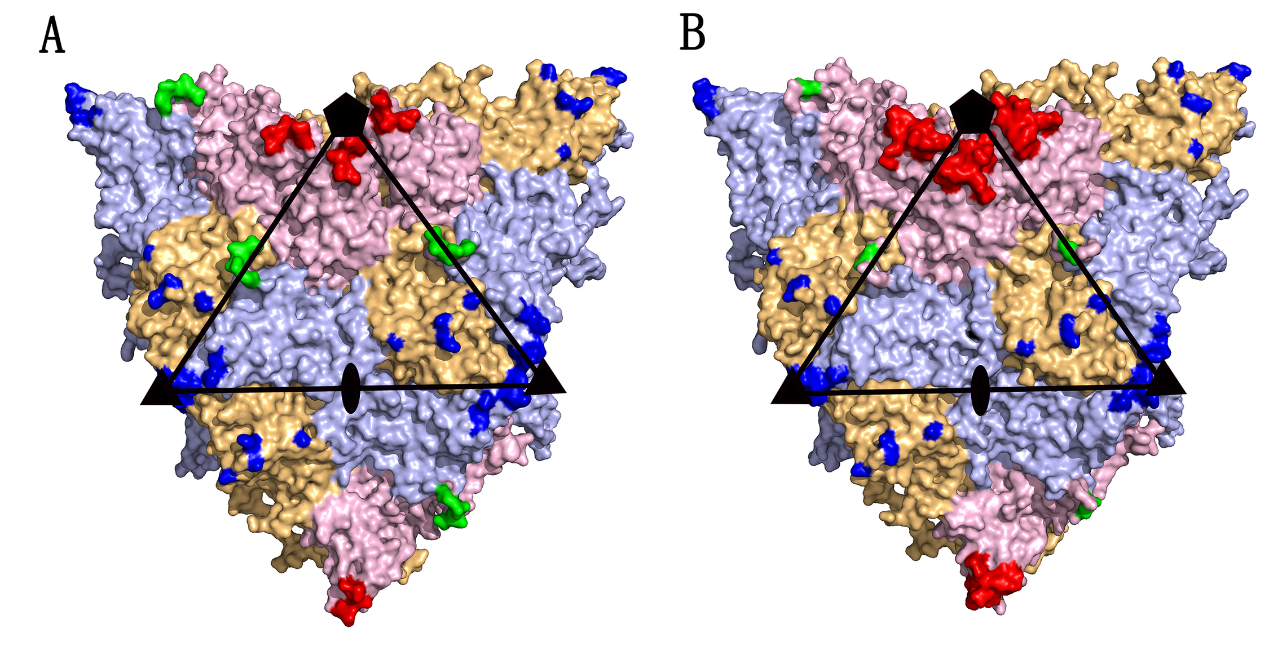


**S3 Fig.** Predicted conformational epitopes of the multiple chains of HPeV1 and HPeV3.

Supplement: S3 Fig — The complete conformational epitopes of the multiple chains of HPeV1 (A) and HPeV3 (B) are presented. The colors and symbols used in this figure are the same as those in Fig 1. (DOCX) [file pone.0247423.s005.docx]
